# First person – Muhammad Dimas Reza Rahmana

**DOI:** 10.1242/dmm.052794

**Published:** 2026-02-02

## Abstract

First Person is a series of interviews with the first authors of a selection of papers published in Disease Models & Mechanisms, helping researchers promote themselves alongside their papers. Muhammad Dimas Reza Rahmana is first author on ‘ Compound design of a patient-derived 3D cell culture system modelling early peritoneal endometriosis’, published in DMM. Muhammad (Dimas) is a PhD student in the lab of Professor Dharani K. Hapangama and Dr Bettina Wilm at University of Liverpool, Liverpool, UK, modelling peritoneal endometriosis using patient-derived cells to study early peritoneal endometriosis formation.


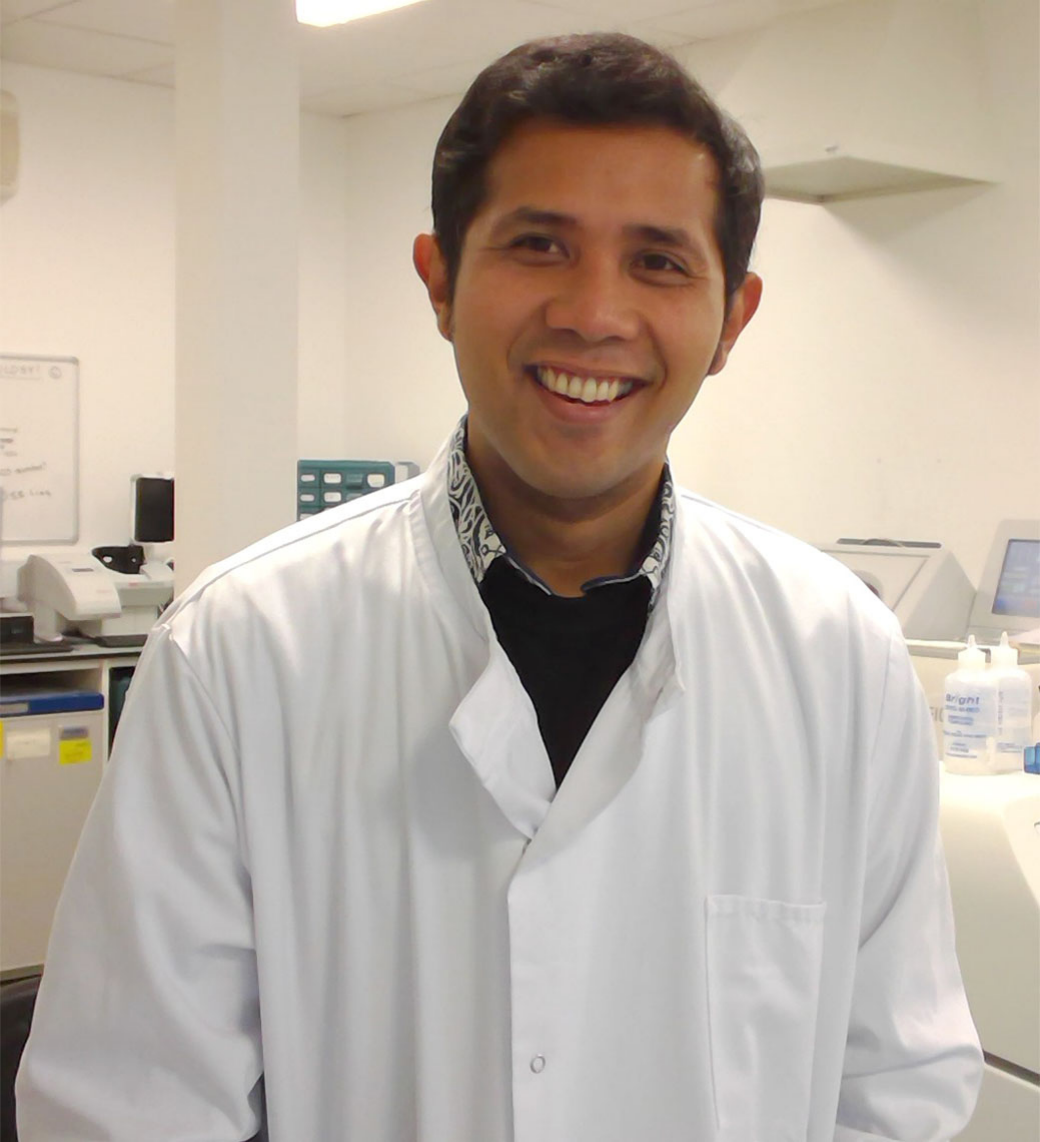


**Muhammad Dimas Reza Rahmana**

**Who or what inspired you to become a scientist?**

One of my favourite experiences in secondary school was the first time I looked through a microscope and saw the microstructure of a thin-sliced onion. It felt like discovering a new universe filled with beautiful structures and patterns. Seeing something invisible to the naked eye for the first time felt almost magical, a memory that has stayed with me ever since and strengthened my interest in microscopy. My enthusiasm deepened further when I studied histology and pathology in medical school. Learning how diseases create complex, observable multicellular microenvironments inspired me to explore cell biology more deeply. The more I learned, the more I realised how many questions remain unanswered. Yet I believe this field always brings together curiosity, discovery and the potential to improve human health. This combination continues to drive my path forward in the scientific field.

Our *in vitro* models would be beneficial for future studies exploring the cellular and molecular mechanisms underlying early peritoneal endometriosis formation

**What is the main question or challenge in disease biology you are addressing in this paper? How did you go about investigating your question or challenge?**

Endometriosis is a chronic inflammatory condition characterised by the presence of abnormally located endometrium-like tissue (the inner lining of the womb) outside the womb. This condition affects approximately one in ten women worldwide and causes chronic pelvic pain that hugely impacting patients' wellbeing. It is believed that retrograde menstrual blood flow allows endometrial fragments to attach to the peritoneum, the thin serous membrane covering the abdominal and pelvic cavities, thereby initiating peritoneal endometriosis formation. However, because endometriosis diagnosis is typically delayed, patient samples are unsuitable to study the early stage of disease development. Meanwhile, the condition cannot be accurately modelled in most laboratory animals due to the absence of a natural menstrual cycle. To overcome this challenge, we collected biosamples from routine laparoscopic surgeries to isolate peritoneal and endometrial cells, which were then used to generate a patient-derived 3D cell culture system. This approach enables detailed investigation of the cellular and molecular mechanisms underlying early peritoneal endometriosis formation.

**How would you explain the main findings of your paper to non-scientific family and friends?**

Our main question is: how does endometriosis get started? If we understand this process, maybe we can identify a way to stop it from happening.

A key step in early peritoneal endometriosis development is the attachment of fragments of the womb lining to the surface of abdominal structures (peritoneum). Our study aims to mimic this process in an artificial environment to help us to understand how endometriosis is initiated. To begin with, we used common byproducts of routine keyhole surgeries, including peritoneal wash fluid and endometrial tissue, as reliable sources of peritoneal and endometrial cells. These cells serve as the primary materials that were expanded to build the model. Microscopic analysis of the resulting peritoneal and endometrial models revealed cellular architectures that closely resembles their structures in the human body, enabling the model to recapitulate the actual cell–cell interaction. Finally, combining both peritoneal and endometrial components enabled the formation of an endometrial–peritoneal interface, where the attachment of cells from both sides is observable. This finding reflects the early process associated to the peritoneal endometriosis development, suggesting the suitability of our model to explore the early peritoneal endometriosis formation.

**What are the potential implications of these results for disease biology and the possible impact on patients?**

Our *in vitro* models would be beneficial for future studies exploring the cellular and molecular mechanisms underlying early peritoneal endometriosis formation. The novel 3D cell culture method described in our study allows the modification of biomaterials to be developed as a more complex system. For example, the cellular components of the model can be expanded by incorporating other cell types, such as immune cells, to examine their roles in the cellular alteration processes involved in early endometriosis. Furthermore, adding menstrual hormones to the culture media can more closely resemble the *in vivo* conditions, supporting further investigation of menstrual hormones involvement during disease progression. Importantly, our cell isolation method relies on biosamples that can be obtained from a single individual patient undergoing routine laparoscopic surgery, without requiring additional or high-risk invasive procedures that undermine patient safety. This approach minimises bias arising from patient-to-patient variability and supports future personalised disease modelling.

**Figure:**
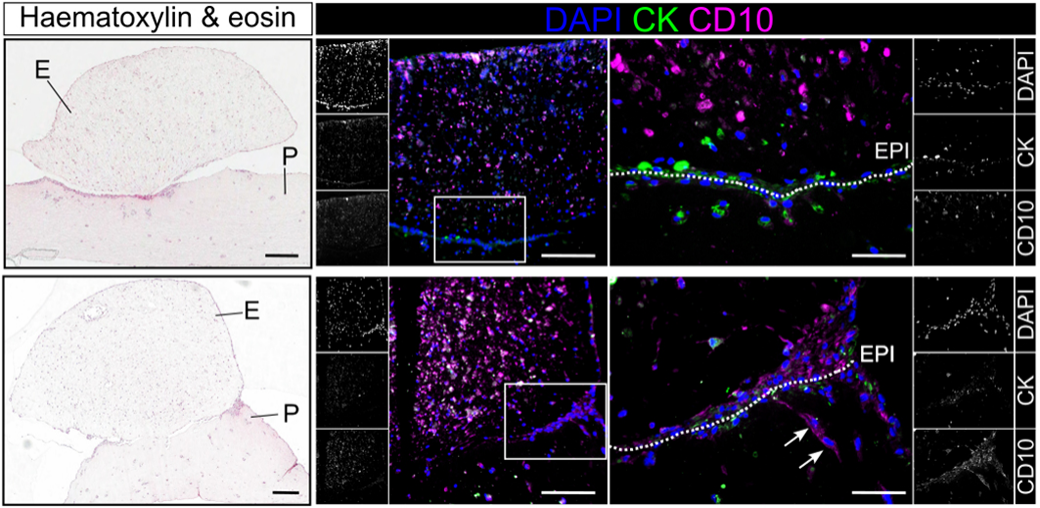
**Histological staining and immunofluorescence of the *in vitro* endometriosis model.** Attachment of endometrial (E) to peritoneal (P) constructs is observed after 3 and 10 days of culture (top and bottom row, respectively). The endometrial–peritoneal interface (EPI; dotted line) was composed of cells expressing cytokeratin (CK) and CD10, with some endometrial stromal cells, the CD10-positive cells, appearing to migrate to the peritoneal compartment (arrows). Scale bars: 200 µm; 50 µm (insets).

**Why did you choose DMM for your paper?**

We chose Disease Models & Mechanisms (DMM) because it is an open-access, peer-reviewed journal that publishes high-quality papers, demonstrating its credibility. As the name suggests, DMM is well suited for our study aiming to develop a disease model. Furthermore, DMM provides valuable references for a wide range of disease modelling techniques that are not limited to a specific disease. This aligns with the utility of our peritoneal model, which can serve as a reference to explore various peritoneal pathologies beyond endometriosis, making DMM an ideal journal for our paper. We believe that publishing in DMM will attract more readers from various backgrounds who are interested in disease modelling and stimulate further research in this field.

**Given your current role, what challenges do you face and what changes could improve the professional lives of other scientists in this role?**

As I move into the final stage of my PhD, the biggest challenge I face is managing my time and deciding what to focus on next. I did not expect my research to lead to numerous interesting scientific questions that are worth investigating further, at the same time as I work on my thesis. Interestingly, this has motivated me to write and publish more extensively. I believe that writing and disseminating research findings is an essential part of scientific practice, as it brings together careful planning, teamwork and a commitment to produce high-quality lab work within targeted timeframes.

**What's next for you?**

The upcoming ‘what's next’ for me is clearly to focus on finishing my thesis. Following my PhD, I plan to pursue a career in academia. This path will give me the opportunity to do what I love and feel passionate about: conducting research and teaching. I believe biomedical science will continue to progress, and contributing to research while teaching this field to students is my ultimate career goal.

**Tell us something interesting about yourself that wouldn't be on your CV**

I am a big foodie! The ‘Come Dine with Me’ and potluck days are some of my favourite activities with my lab group outside of lab work. My kitchen is like my second laboratory, where I love experimenting with different foods. When I have free time, I enjoy going on solo trips to places I have never visited before, mostly to try the local cuisine.
